# Unmet needs in the management of hereditary angioedema from the perspective of Brazilian patients

**DOI:** 10.1016/j.waojou.2024.100992

**Published:** 2024-11-07

**Authors:** Pedro Giavina-Bianchi, Mara Giavina-Bianchi, Raquel de Oliveira Martins, Maria Cristina Fortunato, Ana Claudia Guersoni

**Affiliations:** a Clinical Immunology and Allergy Division, University of São Paulo School of Medicine, Brazil; b Hospital Israelita Albert Einstein, São Paulo, Brazil; c ABRANGHE (Associação Brasileira de Portadores de Angioedema Hereditário), Brazil; d CSL Behring Brazil

**Keywords:** Hereditary angioedema, C1 inhibitor deficiency, Morbidity and mortality, Self-report questionnaire, Unmet needs, Quality of life. angioedemas, Hereditary, Morbidity, Mortality, Self report, Surveys and questionnaires, Health services needs and demand, Quality of life

## Abstract

**Introduction:**

Hereditary angioedema (HAE) is a rare genetic disease characterized by recurrent, potentially life-threatening angioedema episodes. Despite its severity, including the risk of asphyxiation, HAE often remains underdiagnosed. The disease significantly impacts patient quality of life (QoL), leading to anxiety, depression, and avoidance behaviors due to the unpredictable nature of attacks. Understanding the perspectives of patients is crucial for identifying unmet needs in managing this complex condition.

**Objective:**

This study aimed to gather real-world insights from Brazilian patients with C1 inhibitor deficiency HAE to identify their unmet needs and assess their perceptions of the effectiveness of current care in preventing and treating HAE attacks.

**Methods:**

A cross-sectional study utilized a SurveyMonkey questionnaire distributed to HAE patients through ABRANGHE via email. Participants provided informed consent, and their responses were anonymous. The questionnaire, developed with input from experts and patients, covered aspects of HAE diagnosis, treatment experiences, and QoL assessments.

**Results:**

The survey included 178 HAE patients, predominantly female (81%), aged 30–50 years (58%), and college-educated (62%). The most common HAE defect was C1–INH deficiency (53%), followed by HAE-nC1INH (23%), with nearly a quarter unaware of their specific defect. Diagnosis delays were prevalent, with a significant number reporting 13–50 attacks annually (33%) and 15% experiencing more than 50 attacks per year. Laryngeal involvement was reported by 26% of respondents. Most patients (69%) attended regular follow-ups, with 72% on prophylactic treatment and 67% managing acute attacks. The most used acute treatment was Icatibant (49%), followed by pdC1INH (24%). However, confusion regarding medication use persisted, with 45% incorrectly believing that oral medications could effectively treat attacks. Key unmet needs identified included improved access to emergency rooms during attacks (73%), better availability of prophylactic treatment (69%), and enhanced access to specialized care (63%). Patients also emphasized the need for psychological support, increased awareness of HAE, and educational initiatives for patients and healthcare providers.

**Discussion:**

This study highlighted significant challenges in HAE management among Brazilian patients, particularly concerning delayed diagnosis, misconceptions about treatment, and inadequate access to specialized care and prophylactic treatments. The high frequency of emergency room visits underscores the difficulties in managing the disease. The substantial burden of HAE on QoL emphasizes the urgent need for improved physician education, streamlined diagnostic processes, and equitable access to effective medications and specialized care facilities**.** Addressing these gaps is crucial to better support HAE patients, improve diagnostic timeliness, enhance treatment efficacy, and ultimately enhance the overall quality of life for individuals living with HAE.

## Introduction

Hereditary angioedema (HAE) is a rare genetic disease that can be defined as a syndrome characterized by recurrent angioedema that affects the dermis, subcutaneous tissue and mucosa of different parts of the body. HAE has been underdiagnosed despite being potentially fatal and associated with high morbidity.^1^, ^2^, ^3^

HAE can be classified according to its endotype into 2 large groups: HAE with C1 inhibitor deficiency (HAE-C1INH) and HAE with normal C1–INH (HAE-nC1INH). HAE with C1–INH deficiency, the first described and most studied, is caused by a mutation in the SERPING1 gene, which encodes the C1 inhibitor protein, causing increased production of bradykinin, a mediator that promotes vasodilation and increased vascular permeability, leading to angioedema.^4^ HAE-C1INH can be subdivided into Type I, when the protein defect is quantitative, and Type II, when the defect is in its function, with C1INH levels normal or even elevated. In HAE-nC1INH, the C1–INH protein has a normal level and function, and there are 9 other genetic mutations described, such as the factor XII gene mutation.^5^, ^6^, ^7^

The prevalence of type I and II HAE-C1INH is estimated to be approximately 1 in 30,000–50,000 individuals. The diagnosis is based on the patient's history, physical examination, and laboratory tests, which show a reduction in C4 levels in 95% of cases, in addition to assessing the defect in the quantity or function of C1INH.Genetic tests can help in specific situations. As the disease is autosomal dominant, a family history of HAE is a strong predictor for the disease, present in 75–80% of cases.^1^ The diagnosis of HAE-nC1INH depends on the clinical history, genetic testing, and therapeutic trials.^6^ We have little information about the number of HAE patients in Brazil.

The disease burden for patients with HAE is substantial. HAE attacks are unpredictable in frequency and severity and can be fatal if not treated promptly and appropriately. Traumas, stress, traumatic procedures, infections, anxiety, and depression can trigger crises, as well as worsen them, which in turn exacerbates psychological disorders, establishing a vicious cycle. Most patients with HAE live in persistent anxiety between crises, as they present the risk of presenting laryngeal angioedema and asphyxiation. The unpredictable nature of the disease can directly impact patients' daily choices, making them avoid travel, specific hobbies, or social events.^8^, ^9^, ^10^, ^11^ Despite complete physical recovery between attacks, patients often experience ongoing emotional and quality of life (QoL) impairment.^12^, ^13^, ^14^, ^15^

The assessment of health-related quality of life shows patients' perception of the disease, addressing physical, psychological, social aspects, among others.^16^^,^^17^ The outcomes reported by patients are important, allowing the assessment of symptoms as well as the impact, which often cannot be measured objectively by health professionals. This information is collected through qualitative research, such as surveys, covering issues relevant to patients.^18^, ^19^, ^20^ Online surveys have the advantages of including a larger portion of eligible patients, quickly and economically, with data comparable to those obtained by traditional methods. It is becoming increasingly important to know how patients understand and experience aspects related to HAE according to their individual perceptions.

Although the authors, as stakeholders involved in HAE care, perceive that access to specialized medical care, diagnostic tests, and first-line treatments for the disease is precarious in Brazil, making the patient journey burdensome, there is a lack of robust scientific evidence to confirm this scenario. The purpose of this study was to obtain real-world information from patients with HAE through a self-report questionnaire to achieve 2 goals: to obtain real-world information from Brazilian patients with HAE identifying their unmet needs; and to assess if care of patients has been effective in preventing and treating HAE attacks according to their perception.

## Methods

This cross-sectional and descriptive study utilized a structured questionnaire administered to patients with hereditary angioedema (HAE) via the SurveyMonkey platform. The study was approved by the CAPPesq Ethics Committee of the University of São Paulo School of Medicine (CAAE: 36022520.0.0000.0068). After being fully informed, interested patients signed the informed consent form and were included in the study. No financial, or any type of, incentive was offered to the participants.

The questionnaire, developed with input from both experts and patients, aimed to assess and comprehend the journey and the unmet needs of individuals with HAE. We also based our survey on a patient-reported outcome (PRO) tool for the assessment of HAE attacks, which included their management and impact on the lives of patients.^21^

Our questionnaire comprised 30 questions covering demographic details, aspects of HAE diagnosis and treatment, quality of life, and unmet needs, to be completed by patients or their caregivers. The questionnaire (Supplementary material S1) was distributed by ABRANGHE (Brazilian Association of Patients with Hereditary Angioedema; https://www.abranghe.org.br/) via email to its members, ensuring respondent anonymity, from February to June of 2021. Three of the 30 questions and their answers (“What is important to know about the risks of HAE?”; “How and why do you classify an attack as severe?” and “How was your first attack?”) were reported in Supplementary Material S2. The remaining 27 questions were presented across the 3 tables in the manuscript. Collected data underwent review, analysis, and descriptive examination. Subsequently, participants were categorized into 2 groups based on HAE attacks frequency: up to 6 crises annually (group fewer attacks) and 6 or more (group more attacks). Comparative analysis included sex, age, treatment, and disease impact on quality of life.

### Statistical analysis

Descriptive statistics were employed for data analysis. Normally distributed variables were expressed as mean and standard deviation (SD), while categorical variables were presented as counts and proportions (%). For quantitative variables, the nonparametric Student's t-test was utilized. Differences in nominal variables were assessed using chi-square analysis, with the Fisher test applied when appropriate. Reported p-values reflected two-tailed tests, with significance set at p < 0.05.

## Results

The survey was answered by 178 patients with HAE. The majority were female (n = 144; 81%), aged between 30 and 50 years (n = 104; 58%), and had a college degree education (n = 110; 62%). C1–INH deficiency was the most common defect, in 95 individuals (53%), followed by 41 patients with HAE-nC1INH (23%). It is important to notice that almost one-fourth of the patients did not know the subjacent genetic defect in their disease. Demographic characteristics of the patients are described in Table 1.

**Table 1:** Demographic aspects and endotype of patients with HAE (n = 178).

Characteristics		N (%)
**Sex**	Female	144 (80.9)
	Male	34 (19.1)
**Age (years)**	0–12	5 (2.8)
	13–17	4 (2.2)
	18–30	36 (20.2)
	31–50	104 (58.4)
	>50	29 (16.3)
**Educational level**	Elementary/Middle school	16 (8.9)
	High school	51 (28.7)
	College	110 (61.8)
	No education	1 (0.6)
**HAE classification**	HAE-C1INH (Type I)	82 (46.1)
	HAE-C1INH (Type II)	13 (7.3)
	HAE-nC1INH (Type III)	41 (23)
	Don't know	42 (23.6)

HAE: Hereditary Angioedema; HAE-C1INH: HAE with C1–INH deficiency; HAE-nC1INH: HAE with normal C1 inhibitor

We focused our analysis on patients classified with HAE-C1INH types I (n = 82 out of 95, 86%) and II (n = 13 out of 95, 14%). Approximately half of these patients (51%, n = 48 out of 95) reported experiencing their first HAE attack before the age of 8 years. Another 16% (15 out of 95) experienced their first attack between 8 and 12 years of age, and an additional 16% (15 out of 95) between 13 and 17 years of age. Most patients (62 out of 95, 65%) had a confirmed positive family history of HAE. The age at first attack did not correlate with either the number of crises per year or the incidence of laryngeal attacks. The age at first attack also did not correlate with the annual frequency of crises, which could reflect the severity of HAE. We found that 33 out of 48 (69%) patients who experienced their first attack before age 8 had more than 6 crises per year, compared to 29 out of 46 (63%) patients who had their first attack at age 8 or older (p = 0.340). Among patients reporting laryngeal attacks, 15 out of 48 (31%) had their first attack before age 8, while 10 out of 36 (28%) experienced their first attack at age 8 or older (p = 0.3543).

Regarding the time between the first attack and the HAE diagnosis, 51% (48 out of 95) of respondents reported this duration to be over 10 years, 19% (18 out of 95) between 5 and 10 years, 22% (21 out of 95) between 1 and 5 years, and 8% (8 out of 95) less than 1 year. We found a significant difference in diagnostic delay between patients aged 30 years or younger and those older than 30 years. When we divided the time to diagnosis into four groups (<1 year, 1–5 years, 5–10 years, and >10 years) and compared them, a statistical difference emerged (p = 0.003), with younger patients being diagnosed sooner.

The frequency and severity of crises vary significantly among patients with almost one-third experiencing 1–5 episodes annually and another third enduring 13-50 episodes, while 15% report more than 50 crises per year. Treatment also varies, with patients evenly split between treating all, most, or some attacks, and only 8/95 (8%) rarely or never seeking treatment.

The percentage of patients who treat most or all of their attacks is lower among those who use icatibant (47.6%) compared to those who use C1INH (77.8%) or fresh frozen plasma (75.0%). Despite this, the majority avoid hospital visits for treatment, with 58 out of 95 (61%) seeking hospital care less than 20% of the time. This rate was lower for icatibant and C1INH users, 23.8% and 20.9%, respectively, than for fresh frozen plasma users (44%), as the latter is only available in hospitals. The differences were not statistically significant.

During attacks, the most affected areas are the extremities and face (65/95, 68%), followed closely by digestive symptoms (61/95, 64%). Upper respiratory tract involvement (laryngeal) is the most dangerous manifestation, present in 25/95 (26%) of cases, while lower respiratory tract involvement (lungs) is less frequent, found in 12/95 (13%). The majority (74/95, 78%) can sometimes identify prodromic symptoms before an attack.

Regular medical follow-ups are attended by 66/95 (69%) individuals, and 72% (69/95) treat HAE prophylactically, while 67% (64/95) manage acute attacks. There is misunderstanding regarding medication use, with 45% (43/95) believing oral medications like tranexamic acid or attenuated androgens can treat attacks, while some use adrenaline or IV corticosteroids. For acute attacks, Icatibant is the most used first line medication (22/45, 49%), followed by pdC1INH concentrate (11/45, 24%), and fresh plasma (5/95, 5%). Onset of medication action is typically reported after 2 h or more (38/95, 40%), though 45% (43/95) believe it can begin within 30–60 min (Table 2).

**Table 2:** Profile, disease manifestations, and treatments performed in patients with HAE-C1INH, according to amount of crises/year

	Groups regarding number of crisis in one year		Total n (%)
	<6 n (%)	≥6 n (%)	
Questions			
1. **Who is answering the questions? (n = 95)**			
Patient	29 (90.6)	55 (87.3)	84 (88.4)
Caregiver	3 (9.4)	8 (12.7)	11 (11.6)
Total	32 (100)	63 (100)	95 (100)
2. **Patient's sex (n = 95); p = 0.4228**			
Female	24 (75)	52 (82.5)	76 (80)
Male	8 (25)	11 (17.5)	19 (20)
Total	32 (100)	63 (100)	95 (100)
3. **Age (years, n = 95)**			
0-12	3 (9.4)	2 (3.2)	5 (5.3)
13-17	0 (0)	2 (3.2	2 (2.1)
18-30	5 (15.6)	15 (23.8)	20 (21.1)
31-50	16 (50)	36 (57.1)	52 (54.7)
50+	8 (25)	8 (12.7)	16 (16.8)
Total	32 (100)	63 (100)	95 (100)
4. **What is your highest educational level (n = 95)**			
Elementary/Middle school	1 (3.1)	6 (9.5)	7 (7.4)
High school	5 (15.6)	22 (34.9)	27 (28.4)
College	26 (81.3)	35 (55.6)	61 (64.2)
Total	32 (100)	63 (100)	95 (100)
5. **Age of your first HAE attack (years, n = 95); p = 0.4125**			
<8	15 (46.9)	33 (52.4)	48 (50.5)
8-12	3 (9.4)	12 (19)	15 (15.8)
13-17	7 (21.9)	8 (12.7)	15 (15.8)
18-24	4 (12.5)	8 (12.7)	12 (12.6)
25-30	1 (3.1)	0 (0)	1 (1.1)
>30	2 (6.3)	1 (1.6)	3 (3.2)
Not sure	0 (0)	1 (1.6)	1 (1.1)
Total	32 (100)	63 (100)	95 (100)
6. **Family history of HAE (n = 95)**			
Yes, with confirmed diagnosis of HAE	17 (53.1)	45 (71.4)	62 (65.3)
Yes, but no confirmed diagnosis	0 (0)	7 (11.1)	7 (7.4)
No	13 (40.6)	6 (9.5)	19(20)
I don't know	2 (6.3)	5 (7.9)	7 (7.4)
Total	32 (100)	63 (100)	95 (100)
7. **How long between first attack and the diagnosis of HAE (years, (n = 95)**			
<1	2 (6.3)	6 (9.5)	8 (8.4)
1-5	10 (31.3)	11 (17.5)	21 (22.1)
6-10	5 (15.6)	13 (20.6)	18 (18.9)
>10	15 (46.9)	33 (52.4)	48 (50.5)
Total	32 (100)	63 (100)	95 (100)
8. **What is the defect in your HAE? (n = 95)**			
Type I (decreased level)	29 (90.6)	53 (84.1)	82 (86.3)
Type 2 (decreased function)	3(9,4)	10 (15.9)	13 (13.7)
Total	32 (100)	63 (100)	95 (100)
9. **Number of attacks in the past year (n = 93)**			
1-5	30 (100)	0 (0)	30 (32.3)
6-12	0 (0)	19 (30.3)	19 (20.4)
13-50	0 (0)	29 (46)	29 (31.2)
50-100	0 (0)	12 (19)	12 (12.9)
>100	0 (0)	3 (4.8)	3 (3.2)
Total	30 (100)	63 (100)	93 (100)
10. **How many of these attacks are treated? (n = 95)**			
All of them	10 (31.3)	15 (23.8)	25 (26.3)
Most of them	8 (25)	16 (25.4)	24 (25.3)
Some of them	8 (25)	19 (30.2)	27 (28.4)
Rarely or never	1 (3.1)	7 (11.1)	8 (8.4)
Not answered	5 (15.6)	6 (9.5)	11 (11.6)
Total	32 (100)	63 (100)	95 (100)
11. **Regarding the last 10 attacks, how many times did you go to the hospital to get treatment? (n = 95)**			
0	14 (43.8)	17 (27)	31 (32.6)
1	2 (6.3)	5 (7.9)	7 (7.4)
2	8 (25)	12 (19)	20 (21.1)
≥3	7 (21.9)	26 (41.3)	33 (34.7)
Not answered	1 (3.1)	3 (4.8)	4 (4.2)
Total	32 (100)	63 (100)	95 (100)
12. **Which are the commonly affected areas in your body during the attacks? (n = 95)**			
Hands, feet and/or face	21 (65.6)	44 (69.8)	65 (68.4)
Gastrointestinal (abdomen)	18 (56.3)	43 (68.3)	61 (64.2)
Upper respiratory (larynx)	8 (25)	17 (27)	25 (26.3)
Genitals	7 (21.9)	27 (42.9)	34 (35.8)
13. **Can you identify the symptoms before the attacks begin? (n = 95)**			
Yes	15 (46.9)	29 (46)	44 (46.3)
Sometimes	8 (25)	22 (34.9)	30 (31.6)
No	1 (3.1)	2 (3.2)	3 (3.2)
Not answered	8 (25)	10 (15.9)	18 (18.9)
Total	32 (100)	63 (100)	95 (100)
14. **Do you do medical follow ups for HAE? (n = 95)**			
Yes	22 (68.8)	44 (69.8)	66 (69.5)
No	3 (9.3)	9 (14.3)	12 (12.6)
Not answered	7 (21.9)	10 (15.9)	17 (17.9)
Total	32 (100)	63 (100)	95 (100)
15. **Do you treat for HAE? (n = 95); p = 0.2 for attacks treatment and p = 0.33 for prophylaxis treatment**			
Yes, but only in attacks	8 (25)	12 (19)	20 (21.1)
Yes, but only prophylactically	17 (53.1)	27 (42.9)	44 (46.3)
Yes, both attacks and prophylactically	4 (12.5)	21 (33.3)	25 (26.3)
No	3 (9.4)	3 (4.8)	6 (6.3)
Total	32 (100)	63 (100)	95 (100)
16. **How do you treat the HAE attacks? (n = 95)**			
Intravenous medication	6 (18.8)	12 (19)	18 (18.9)
Subcutaneous medication	7 (21.9)	18 (28,6)	25 (26.3)
Oral medication	15 (46.9)	28 (44.4)	43 (45.3)
Fresh plasma	2 (6.3)	3 (4.8)	5 (5.3)
Not answered	2 (6.3)	2 (3.2)	4 (4.2)
Total	32 (100)	63 (100)	95 (100)
17. **Which is the medication for attacks? (n = 49)**			
Fresh plasma	2 (4.1)	3 (6.1)	5 (10.2)
pdC1INH concentrate	5 (10.2)	6 (12.2)	11 (22.4)
Icatibant	7 (14.3)	17 (34.7)	24 (49)
Tranexamic acid	4 (8.2)	0 (0)	4 (8.2)
Adrenalin	1 (2)	1 (2)	2 (4.1)
Intravenous or oral corticosteroids	0 (0)	3 (6.1)	3 (6)
18. **After taking the medication for an attack, how long does it take before you begin to feel relief from the symptoms? (n = 95)**			
Immediately	4 (12.5)	2 (3.2)	6 (6.3)
30–45 min	5 (15.6)	18 (28.6)	23 (24.2)
Around 60 min	8 (25)	12 (19)	20 (21.1)
2 or more hours	12 (37.5)	26 (41.3)	38 (40)
Not answered	3 (9.4)	5 (7.9)	8 (8.4)
Total	32 (100)	63 (100)	95 (100)
19. **Which is the impact of HAE in your quality of life? (n = 95); p = 1.00**			
High	29 (90.6)	58 (92.1)	87 (91.6)
Low	3 (9.4)	5 (63)	8 (8.4)
Total	32 (100)	63 (7.9)	95 (100)

**Fig. 1:**
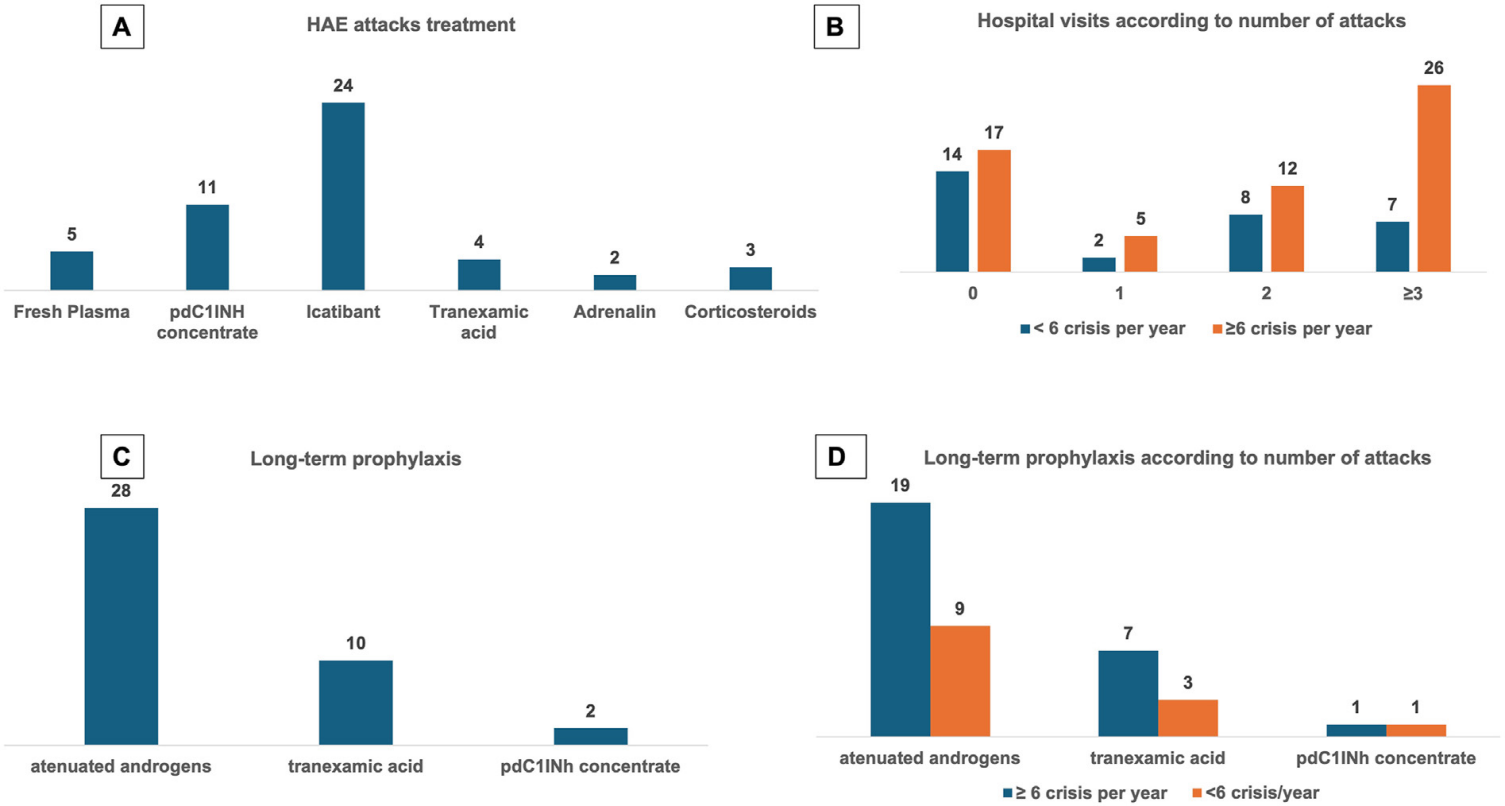
Treatments used on attacks (A), long-term prophylaxis(B) and comparison of hospital visits for treatment(C) and prophylactic treatment(D) used by patients with <6 or ≥ 6 attacks per year

Two groups of patients were established based on the frequency of angioedema attacks, with “fewer attacks” (n = 30) defined as those with up to 6 episodes per year and “more attacks” (n = 63) with 6 or more episodes per year (Table 2). We tested the 2 groups searching for significant differences in 4 topics: sex, age of the first attack, treatment used in attacks, and use of prophylactic medication (Fig. 1). There were no statistical differences in the comparison of the groups <6 attacks/year and ≥6 attacks/year regarding any of these variables. We repeated the analysis, now dividing the patients into two groups using 12 attacks per year as the criterion for division (<12 attacks/year and ≥12 attacks/year). Once again, we did not find statistically significant differences between the 2 groups. There was a trend of women having a higher rate of more than 12 attacks per year than men (p = 0.07).

The second part of our study assessed patients’ expectations and knowledge about HAE. Most patients (81/95, 85%) believe they know what to do in an emergency during their attacks. All patients expressed a desire to have medications for crises at home, but one-third (33/95) do not feel capable of administering IV medications. Moreover, 7% (7/95) have concerns about administering subcutaneous medication. Regardless of whether the medication is IV or SC, all patients agreed that it is important to receive training on how to administer them.

The burden of HAE was classified as high by 87/95 (92%) of patients. Among their unmet needs, the main desire was easy access to emergency rooms during attacks (69/95, 73%), followed by access to prophylactic treatment (66/95, 69%) and specialized physicians (60/95, 63%). Patients emphasized the need for medications at home, highlighted the lack of disease knowledge among generalists in emergency rooms, and stressed the need for psychological support, more publicity about HAE, and more options for prophylactic treatments. They also mentioned the need for information on where to find specialized care centers, associations, and access to treatment in their vicinity. Support and educational programs can be beneficial, providing patients with instructions about access, mode of use, and adverse effects of medications, as well as educating emergency care professionals about their disease. Additionally, patients need information and instructions on how to avoid crises, recognize the signs and symptoms of HAE, understand treatment options, and locate specialized centers. (Table 3).

**Table 3:** Knowledge and expectations about the HAE's management

Question	n (%)
1. **Do you know what to do in an HAE emergency? (n = 95)**	
Yes	81 (85.3)
No	13 (13.7)
Not answered	1 (1.1)
2. **Do you believe it is important to have the attacks' medication at home? (n = 95)**	
Yes	95 (100)
No	0 (0)
3. **Would you be able to administer an intravenous medication by yourself? (n = 95)**	
Yes	62 (65.3)
No	33 (34.7)
4. **Would you be able to administer a subcutaneous medication by yourself? (n** = **95)**	
Yes	88 (92.6)
No	7 (7.4)
5. **Do you believe it is important to have training about how to apply intravenous or subcutaneous medications for HAE? (n** = **95)**	
Yes	95 (100)
No	0 (0)
6. **What do you believe to be important for your HAE control? (n = 95)**	
Easy access to emergency room in attacks	69 (72.6)
Easy access to prophylactic treatment	66 (69.5)
Easy access to my HAE doctor	60 (63.2)
To have the medication at home	7 (7.4)
To have more physicians in the emergency room with knowledge regarding HAE	4 (4.2)
To have psychological support	2 (2.1)
More mediatic HAE exposition	2 (2.1)
More options of prophylactic medications	1 (1.1)
7. **Which of the following information is important for HAE patients? (n = 95)**	
To know how to access treatment near by	76 (80)
To know where the specialized care is located	65 (68.4)
To know the specialized physicians in the area	48 (50.5)
To know the patient HAE associations	41 (43.2)
8. **Which of the following topics would be important in a support program for patients? (n = 95)**	
Instructions how to perform injectable medications	73 (76.8)
Instructions on how to obtain the medications	66 (69.5)
List of the specialized centers	59 (62.1)
Instructions how to avoid attacks	56 (58.9)
Instructions on HAE signs and symptoms	39 (41.1)
To train emergency room professionals about HAE	4 (4.2)
Juridic support to obtain medications if necessary	1 (1.1)
Governmental support	1 (1.1)
To inform about adverse effects of HAE medications	1 (1.1)
To instruct which medications to avoid in HAE	1 (1.1)
To have a channel for the patient to call when in an emergency room	1 (1.1)
To facilitate the access to medications	1 (1.1)

## Discussion

Our survey included 178 individuals with HAE. Given that Type I and Type II HAE exhibit similar signs, symptoms, progression, and management, we decided to analyze these 2 types together in detail, encompassing 95 patients. Although Type III HAE was relatively common in our study, it has distinct clinical features and management approaches. Therefore, we opted not to combine it with Types I and II. Unfortunately, nearly a quarter of our patients were unaware of their specific HAE type, so they could not be included in any other assessment. This lack of knowledge regarding their HAE type could be due to factors such as limited access to diagnostic services or insufficient patient education. A similar result was found in another study.^15^

Among the 95 individuals with C1INH deficiency, 86% had Type I and 14% had Type II. This result aligns with existing literature, which indicates that Type I occurs in 80–85% of cases and Type II in 15–20%, adding robustness to our survey.^10^^,^^11^ Although HAE is expected to have an equal number of cases in men and women, since it is an autosomal dominant disorder,^4^ our survey consisted of 80% women. This disparity might be due to multiple organic or behavioral reasons. On one hand, men typically seek medical attention less frequently, attend follow-ups less often, and may be less inclined to respond to surveys than women. On the other hand, estrogen is associated with a worse progression of the disease.^1^^,^^2^^,^^8^

Many individuals with HAE experience a similar disease course, with most showing the first signs during childhood and worsening during puberty.^8^^,^^10^ In our survey, clinical symptoms began in 51% of patients before the age of 8, and in another 32%, between 8 and 17 years old, corroborating Bork's case series finding that 50% of cases begin before the age of 10.^11^

The time between the first attack and diagnosis was more than 10 years for 51% of the individuals. This prolonged period between disease onset and diagnosis increases the risk of death and disease-related morbidity.^8^^,^^12^^,^^22^^,^^23^ Long delays in diagnosis occur worldwide. In Spain, a 2005 study showed that the average time to diagnosis was 13.1 years.^10^ A survey of physicians in the United States reported that fewer than 38% of HAE patients were diagnosed within 1–3 years of their first attack.^11^ Several factors contribute to this delay: first, the rarity of the disease; second, the symptoms can easily be misdiagnosed as other more common conditions; third, the lack of knowledge and recognition of HAE by general physicians, including in the emergency rooms. This latter issue was strongly supported by our Brazilian survey, highlighting the need to educate emergency room medical staff about HAE, as they often are unaware of the disease and skeptical of patient reports. Better actions must be taken by physician and patient organizations, the government, and industry to improve this situation. However, this issue also appears to be present in other countries. In an online survey from 2004, 63 patients with HAE reported an average of 4.7 emergency room visits per year, with nearly 21% receiving treatment for anaphylaxis in the emergency department in the United States,^12^ mirroring our finding of 1 patient in our study receiving adrenaline for HAE attacks. However, the results of the present study show that despite all the difficulties, we are making progress, as patients aged 30 years or younger are being diagnosed earlier.

HAE affects the skin and submucosa, including the submucosa of the upper respiratory tract, oropharynx, and gastrointestinal tract, as corroborated by our study. The duration of attacks varies from 2 to 5 days before improving spontaneously.^13^ If the diagnosis is incorrect, the treatment will likely be ineffective and inappropriate. During the time without an HAE diagnosis, a life-threatening attack may occur. Making the correct diagnosis will enable patients to be educated about managing their condition in daily life, including identifying and avoiding triggers, handling acute attacks, and responding to urgent situations such as airway obstruction. These factors were highlighted as highly relevant by patients in our survey. Screening family members for HAE may also help avoid unnecessary tests and procedures. In our survey, 72% of the cases had a family history of HAE, with 65% confirmed by laboratoty tests. Therefore, asking patients about family history might be a simple and important question to ask in both emergency rooms and primary care settings.

Our second goal was to assess the effectiveness of the current management of HAE. Regarding the treatment of attacks, excluding those that are not appropriate for HAE, such as adrenaline and intravenous corticosteroids, and others meant for prophylaxis, we observed that Icatibant was the most used medication (49%), followed by pdC1INH concentrate (24%), and fresh plasma (11%). Therefore, 3/4 of the patients used first-line medications to treat HAE attacks.

We assessed the number of attacks per year and evaluated variables such as age, sex, prophylactic treatment, and quality of life. We found that 65 out of 95 (68%) individuals experience 6 or more attacks per year. The division of patients by the number of attacks per year, using six attacks per year as a criterion, was a choice based on the authors' experience and the literature. There is no universally recognized number of attacks per year that is considered "acceptable" for a patient to have. Ideally, it would be zero, but we consider that a patient should not have 6 or more attacks per year, as this would be associated with a significant loss of quality of life. In the present study, there was no associations between the number of attacks and the variables tested.

This suggests that the prophylactic treatment currently used by these patients has not being effective. If 68% of these patients have follow-ups with a specialist, what is going wrong? Are the patients misinformed? Despite the high efficacy and safety of long-term prophylactic drugs, do patients lack access to the proper medications? Consequently, 35% of these patients visit the emergency room 3 or more times per year.

In Brazil, access to first-line medications for the treatment of HAE attacks (icatibant and intravenous C1 inhibitor) is limited both at home and in hospitals. Often, patients avoid emergency rooms because they know they will encounter doctors unfamiliar with their disease, and hospitals that carry these medications are rare. The few patients who have these medications at home only use them in the most severe attacks because they know that replacement will take a long time.

The same situation occurs with long-term prophylaxis. The way long-term prophylaxis is being carried out in Brazil has not been effective. The patients included in the study are followed by specialists who are up to date with the treatment of hereditary angioedema. However, although first-line medications (Lanadelumab, subcutaneous C1INH concentrate, and berotralstat) are registered and available in the country, accessing them is limited. In the past, we used up to 600 mg of danazol and managed to control the vast majority of our patients. Currently, in accordance with the latest guidelines, we usually prescribe the maximum dose of 200 mg of danazol, which is insufficient to control a significant percentage of patients. Attenuated androgens have been used since the 1970s to prevent HAE attacks.^14^ A retrospective study of 118 patients using danazol for long-term HAE prophylaxis reported an 84% reduction in the average number of attacks per year compared to the pretreatment period. During danazol therapy, 24% of patients had no symptoms, 22% experienced ≤1 attack per year, and 27% had between 1 and 5 attacks annually. However, 14% of patients experienced at least 11 attacks per year despite treatment, and 30 patients withdrew from the study due to side effects.^15^ In another study, 62.4% of patients were using long-term prophylaxis, including 34.4% using androgens, yet an average of 12.5 moderate attacks were reported in the six months prior to the survey.^24^

The lack of control over HAE significantly impacts quality of life, as described in various articles.^7^^,^^9^^,^^16^ One study reported moderate to severe anxiety in 38.0% of patients and depression in 17.4%, with the severity of these conditions correlating with reduced quality of life and productivity.^24^ Our findings indicate that 92% of participants experience a high burden from HAE. Effective HAE management should aim to restore normal quality of life for patients.^24^

There appear to be numerous unmet needs in HAE care in Brazil: enhanced education for primary care physicians and allergists/immunologists regarding HAE screening and diagnosis; increased awareness of the disease among emergency care professionals; expedited diagnosis procedures; patient education on managing HAE in daily life, such as identifying and avoiding triggers, preparing for acute attacks, and responding to emergencies; and better accessibility to first-line medications for both attack management and prophylaxis, as well as specialized care.

The limitations of this study include the relatively small sample size, which may limit comparisons between subgroups and the generalization of the results to the broader population with hereditary angioedema in Brazil. The patients completed the questionnaires independently, introducing the possibility of misunderstandings or inconsistent interpretations of the questions and answer options, which may affect the reliability of the responses. Reliance on self-reported experiences of patients also introduces the risk of recall bias, particularly concerning the timing and frequency of attacks or emergency room visits. Additionally, while many patients reported using androgens for long-term prophylaxis, the questionnaire did not capture the specific doses they were taking, which is significant since the efficacy and side effects of androgens are dose dependent. Lastly, the cross-sectional design of the study provides only a snapshot in time, limiting its ability to assess the long-term effectiveness of treatments or changes in disease management.

## Conclusion

Our survey reveals multiple aspects of HAE patients, including demographics, personal history, family history, management, quality of life, and expectations. It also highlights the shortcomings of the Brazilian health system in managing HAE, particularly in long-term prophylaxis. However, despite these challenges, the results of the present study indicate progress, as patients aged 30 years or younger are being diagnosed earlier. We hope that our survey can raise awareness among the general population, government policymakers, physician associations, and patient advocacy groups about the unmet needs of individuals with HAE. By doing so, we aim to prevent avoidable deaths, reduce unnecessary procedures, and enhance the overall quality of life for these patients.

### Disclosure of use of artificial intelligence tools

During the preparation of this work the authors used Chat GPT in order to improve language and readability. After using this tool, the authors reviewed and edited the content as needed and take full responsibility for the content of the publication.

## Ethics statements

The study was approved by the Ethics Committee of the University of São Paulo School of Medicine (CAAE: 36022520.0.0000.0068), and all the data contained in it is available from the authors. After being fully informed, interested patients signed the informed consent form and were included in the study. No financial, or any type of, incentive was offered to the participants.

## Consent for publication

All authors wrote, read, approved, and consented to the publication of this manuscript.

## Funding

The study was done at the Clinical Immunology and Allergy Division of the University of São Paulo School of Medicine, Brazil, in 2024, using own Department funding.

## Declaration of competing interest

The authors ACG and MCF are employees of CSL Behring. The author MGB has financial and conflicts of interest to disclosure with CSL Behring. The author PGB has financial and conflicts of interest to disclosure with CSL Behring, Takeda/Shire, and Pint Pharma, but he did not receive any financial compensation to develop the present study. The author ROM has no financial or conflicts of interest to disclosure.

All authors participated in the design and the development of the present study.
