# Supplementary material for: Unmet needs in the management of hereditary angioedema from the perspective of Brazilian patients
Source: World Allergy Organ J. 2024 Nov 7;17(11):100992. doi: 10.1016/j.waojou.2024.100992 (PMC11582446; doi:10.1016/j.waojou.2024.100992)
Supplement: Multimedia component 1 [file mmc1.docx]

**Supplementary Material S1**

**Questionnaire:**

1. The person answering is the:

Patient

Care giver

1. Patient's sex:

Feminine

Masculine

1. What is your age range (if you are the patient) or the age of the patient you care for? (years)

0-12

13 to 17

18-30

31-50

≥50

1. What is your degree of education:

Elementary School

High school

University education

I have no study

1. How old were you (or the patient) when you had your first HAE attack?

< 8

8-12

13-17

18-24

25-30

>30

I am not sure

1. Are there people in the family (of the patient) with the same symptoms?

Yes with confirmed diagnosis of HAE

Yes, with no confirmed diagnosis of HAE

No

I don't know

1. How long after the first angioedema attack was the diagnosis of hereditary angioedema (HAE) made?

<1 year

1-5 years

5-10 years

>10 years

1. Do you know the classification of your (or your patient's) hereditary angioedema?

Type 1 (low quantity of C1 inhibitor)

Type 2 (low function of C1 inhibitor)

Type 3

I don't know

1. How many attacks have you or the patient had in the last year? (open question)
2. How many of these crises are treated?

All of them

The majority

Some

Rarely or never

1. Of the last 10 crises you had, how many did you need to treat in the hospital? (open question)
2. Which area(s) are typically affected during crises?

Hands, feet, and/or face

Gastrointestinal/ Abdomen

Upper respiratory/Larynx

Lower respiratory/Lungs

Genitals

1. Can you identify the initial symptoms before a crisis happens?

Yes

Sometimes

At the

1. Do you have regular medical follow-up for EAH?

Yes

No

1. Do you (or the patient) use any treatment for HAE? If yes, which type(s)?

Yes, only for crises

Yes, only prophylactic

Yes, for crises and prophylactic

No

1. What type of treatment(s) do you use in an HAE crisis?

Intravenous medication

Subcutaneous medication

Oral medication

Fresh plasma

1. Which is the medicine used and in what dosage? (open question )
2. When you use the treatment in crisis, how long does it take to start feeling relief after administration?

Immediately

30-45 minutes

Close to 1 hour

≥2 hours

1. How much do you believe that hereditary angioedema impacts your quality of life?

Much

Little

1. If you have an emergency, do you know what to do?

Yes

No

1. Do you think it’s important to have a medication that you can take at home in a crisis?

Yes

No

1. Do you think you could administer an intravenous medication (into a vein) at home?

Yes

No

1. Do you think you could apply a subcutaneous medication at home?

Yes

No

1. Do you think it is important to receive training on how to use the medication at home (intravenous or subcutaneous)?

Yes

No

1. Which of the points below do you consider to be most important for patients with HAE?

Easy communication with the HAE specialist doctor

Easy access to emergency services in crisis

Access to HAE prophylactic treatment

Other, please describe

1. Which are important informations for patients with HAE?

To know where the specialized care is located

To know how to access treatment near by

To know the specialized physicians in the area

To know the patient HAE associations

1. If there is a patient support program from the company that provides HAE treatments, which services do you believe are important? Select one or more alternatives:

In the case of using injectable products, instructions on the preparation, storage and application of the medication

To have information about how to get the medicine my doctor prescribed for me

A list of specialized service centers

Information about signs and symptoms of HAE

Information on how to avoid HAE attacks

Other information that you consider relevant, please describe

1. What do you consider important to know about the risks of HAE? (open question)

Answers:

| - What triggers a crisis, I think is very important. |
| --- |
| - About when to treat the crisis so it doesn't get worse. |
| - All. |
| - Everything about risks is important, until about 6 years ago I didn't know that was the case. |
| - Not getting the necessary medications for the patient. |
| - How to access medicines. |
| - Mainly medical procedures that can cause swelling in the airways. It is also important to know that this is not an allergy and cannot be treated with anti-allergens. |
| - Long-term organ damage. |
| - Foods. |
| - I can't say. |
| - How to proceed in serious situations. |
| - Life risks. |
| - Have medical follow-up. |
| - I have little information. |
| - I believe that every patient should be well-informed about the disease and the treatment. |
| - What triggers crises and what medication to treat them. |
| - The effect of medication on the rest of your health. |
| - You can never wait. You need to act, be prepared (medication). |
| - All. |
| - All risks are important to assess, and such a crisis could be fatal! |
| - That it can be confused with allergies, that it can kill in a larynx crises, and that contraceptives make things much worse. |
| - Everything, because there is little information even among health professionals. |
| - Taking care of surgical and dental procedures, repetitive movements as trauma is what usually triggers my crises. |
| - Know how to recognize when the crisis is severe and know how to proceed, how to seek help. |
| - Side effects of the medicine and prevention. |
| - The risk of suffocation and difficult pains |
| - That it is a dangerous disease, which can lead to death, and the patient needs to know about it and treat it right from the beginning and most importantly, do prophylaxis. |
| - I take everything into account, area of the edema, how it evolves and risks, to treat it urgently. |
| - All. |
| - All information about the disease, forms of control and also the risks are extremely important. |
| - How to avoid glottis edema. |
| - Medication. |
| - Know how to assess the severity of the crisis |
| - Knowing how to assess severity |
| - What measures and medications are recommended when a crisis begins |
| - What are they and how can they be avoided. |
| - It is important to know the places where you can have a crisis and always be on the lookout, especially in the throat. |
| - About the care you should take because not all medication works |
| - Information about the disease, prophylactic and emergency medications, how to identify what triggers crises, how to treat them and what to do in an emergency. |
| - That doctors get to know about the disease. |
| All. Medicines, food. |
| - As soon as your throat starts to swell, run to the hospital. |
| - I think all patients should have access to information, treatment, control and risks of the disease. Especially in crises that can lead to death. |
| - That if I take medical action at the beginning of the crisis, I can avoid the progression to glottal edema and avoid intubation. |
| - To know how to correctly inform the doctor if you need to consult. |
| - When is it a serious risk? |
| - Knowledge about the disease, treatments and remedies |
| - It is important to know the risks so that we can prevent or treat them as quickly as possible. |
| - Prevention |
| - Edema in the respiratory tract and how to react |
| - Every mild crisis can become a serious/very serious crisis in a very short time. Therefore, it is very important that someone close to you is aware of the situation and monitors the patient. There is also an insecurity regarding health professionals who almost never know about this disease and end up treating it in inappropriate and even harmful ways. |
| - To have specific treatment aimed at preventing crises. |
| - Everything related to risks |
| - That a fatal crisis can happen at any time. |
| - Medicines that cannot be used |
| - How to help reduce symptoms and how to treat if you go to the emergency room |
| - Understanding the disease as many doctors do not know about it, sometimes we, patients, have to tell them what to take in a crisis at the hospital |
| - Risk of glottis edema. |
| - That is a disease that can be fatal. |
| - Triggers and emergency medications. |
| - I know almost everything. |
| - Which medication will be efficient to treat (end) the crisis. |
| - About glottis swelling, and how to help |
| - Glottis edema |
| - The difference between Allergic and Hereditary Angioedema |
| - For me today, nothing. I try to take care of my mind mainly. |
| - About new treatments other medications if possible |
| - All. |
| - To prevent death. |
| - As many doctors don't know, they can perform a wrong procedure. |
| - Swellings in the glottis. And surgical processes in general. |
| - Diagnosis, affordable treatment. |
| - It is important to pay attention to the beginning of the crisis and treat |
| - What can trigger it and what risks are |
| - For sure |
| - Know how to treat |
| - What are the main triggers and the right procedure |
| - The types of treatment available |
| - I consider everything important |
| - Identify crises, as emergency doctors never know what they are. |
| - It is important that more doctors know about the disease because my son was treated several times with the wrong diagnosis. |
| - I've read it somewhere, but I'd like to know if I'm at risk of swelling my brain and what the consequences are? |
| - Extremely important to avoid death |
| - How to avoid crises and how to treat them |
| - Whatever it takes. |
| - Yes |
| - Possibility of treatment |
| - it's good to know so we can stay on top of the subject. |
| - Swellings that can lead to death. There is not an impactful disclosure about the risks of the disease. |
| - That can lead to death |
| - Know the facts/activities that trigger crises. Carrying medication with me is mandatory to have a peaceful/safe life |
| - Be careful with any other medication to avoid triggering crises. It is important to have doctors who know the disease. |
| - That is a disease that can lead to death. That is a disease that is often misdiagnosed. That there is poor hospital structure and knowledge about the disease. It is difficult to access medicines, both prophylactic and high cost. |

1. When do you consider a crisis serious? Why? (open question)

Answers:

| - When it affects the airways, when it interferes with my work rhythm, my quality of life. |
| --- |
| - In the upper area: chest, neck, symptoms of difficulty breathing or swallowing |
| - Edema in the glottis or mouth. |
| - When it attacks the throat because I'm afraid it will close during sleep, and I’ll die. |
| - Glottis edema, gastrointestinal edema, and the last crisis that according to my patient reported a swollen head. |
| - When it affects the throat. |
| - When the swelling is significant, causing considerable discomfort and being visually noticeable. Sometimes small areas of my body swell, like a single. finger, but in this case, I don't consider it a crisis, as it is a minor event. |
| - When it reaches the airways, it can be fatal and causes a lot of suffering. And when it affects abdominal organs, because I feel very severe pain and I am afraid and uncertain about the consequences. |
| - They are all horrible. |
| - When more than one part of the body swells. Because it often stops me from doing my work and breaks me down psychologically, showing that I will never be cured. |
| - When it occurs on the face, it generally reaches the throat. |
| - When it hits the face, there is a risk of reaching the glottis. |
| - Swelling of the glottis as it can be fatal. |
| - Shortness of breath and abdominal pain, social problems and daily activities begin to occur. |
| - Larynx. Asphyxiation. |
| - When it occurs in the thoracic region. Because is it difficult to breathe. |
| - When it takes more than a day to get better. |
| - Even after a high dose of medication, it still recurs. |
| - Face due to glottis, and abdominal due to dehydration. |
| - I've had glottis edema 3 times and it worries me a lot. When I am without medication (ladogal / danazol) the edema appears again and again, I believe with more intensity due to the nervousness of not having the medication. I get very anxious and worried without medication. |
| - Risk of death or severe abdominal pain. |
| - When swelling in the face, throat. |
| - glottis edema is very severe, but abdominal crises are quite disruptive, as intravenous analgesics are generally needed to alleviate the pain, which makes working impossible. |
| - Abdomen and glottis. Abdomen because it hurts a lot and is very disabling and glottis because it is life-threatening. |
| - Swelling of the glottis and of the intestinal loops, I even fainted from the pain. |
| - On the face or private parts. On the face because it is close to the throat and private parts due to the pain and difficulty in performing functions. |
| - When even medicated, the crisis does not regress. |
| - Edema on the face and throat. |
| - Glottis edema. I've been at risk of death 5 times. |
| - When it hits the mouth, uvula, pharynx. Due to the risk of suffocation if it hits the glottis |
| - When the glottis or tongue swells |
| - When it is on the face, abdomen, throat and chest |
| - Face, glottis and neck |
| - When your face swells |
| - Facial edema, especially in the mouth, due to the risk of glottis edema. |
| - When it is in the throat region |
| - When it is on abdominal or on the face, that can go down to the throat. |
| - I rarely have it in my stomach, but whenever I do I get quite scared. My biggest fear is that I'll get it in my throat and be short of breath. Thank God, I never had it. |
| - When your throat swells, you can't breathe or eat. |
| - When it is internal swelling, face and throat. Because they can progress to laryngeal edema, which in my understanding is the most serious |
| - When I have edema in the glottis, because it can lead to death |
| - On the face for fear of reaching the throat. In the stomach/intestine due to the fear of being misdiagnosed in the hospital. |
| - When it goes to the throat, because I'm afraid of blocking my breathing |
| - When it was in the throat, as I couldn't swallow and felt like vomiting, causing choking and shortness of breath |
| - When it affects the face going down to the glottis, because it makes breathing difficult |
| - Abdomen. Crises in the abdomen cause almost unbearable pain, diarrhea and dizziness. |
| - When it occurs on the face and throat. |
| - When the face swells too much, as it is close to swelling the glottis (I think) |
| - Swelling in the larynx and possible death from asphyxiation. |
| - When the pain is intense and I realize that I won't get better with the medications I have at home and I need to go to the hospital |
| - On the face, because of the risk of hitting the glottis. I even had a tracheostomy. |
| - Crises in the abdomen (a lot of pain, fainting and impossible to treat at home) |
| - For me, a serious crisis is when the swelling is internal (uterus, ovary, intestine and stomach). External crises, no matter how bad they are, don't make me go to the hospital. However, I am totally or partially disabled. |
| - When there is swelling in the neck, or acute pain in the abdomen. |
| - In the glottis, because I can't breathe and speak. |
| - Abdominal crises are serious and make me faint from the pain when they are very strong. Crises that affect the respiratory tract. Every crisis can become fatal considering that it can affect an organ or region that had never been affected before. |
| - Internal swelling. |
| - In abdominal crises, because I have a lot of pain, diarrhea, vomiting and nausea. |
| - In face and throat edema, due to the respiratory tract, I have already had 2 deaths in the family for this reason. |
| - When it affects the throat |
| - A crisis that leaves me unable to carry out normal daily activities. |
| - When swelling causes pain and makes it difficult to carry out daily activities. |
| - When it's in the throat. |
| - The serious crisis is on the face and throat, as it can impede the passage of air. Crises that occur in the stomach are very painful and debilitating. Swelling in the hand, foot, leg and the like is quite uncomfortable. |
| - When it hits the larynx or stomach. |
| - Due to pain. |
| - All because they all do a lot of harm. |
| - Glottis edema, because I had to fight to live. |
| - Larynx because it can take over in a short time. |
| - When I am unable to continue my routine. |
| - In a critical region, but I learned how to administer medication and when there is a major crisis, I do it, which is why we no longer go to the hospital. |
| - When it leaves me incapacitated, with very severe abdominal pain. |
| - When the abdomen swells, in addition to being very painful, almost unbearable, several edemas appear in different places, but one at a time. So, this crisis takes days to pass. |
| - When it affects the face due to suffocation. |
| - When I dehydrate and have hypotension. I'm afraid I'll go into shock. |
| - Almost every time. |
| - Abdominal, chest and airways. |
| - When it affects respiratory function (larynx and pharynx), as I feel short of breath. |
| - Abdominal, because I can't walk, I can barely breathe because of so much pain, and glottis, it is horrible. |
| - Pain and difficulty swallowing. |
| - Only when it is on the face and there is a possibility of glottis edema. |
| - When it affects the face and then goes down to the throat because it is almost impossible to breathe. |
| - The glottis, tongue, esophagus, the inner part of the rectum. |
| - Abdomen and throat. |
| - Life-threatening glottis edema. |
| - When it hits the throat. |
| - The possibility of glottis edema. |
| - On the face and pharynx because it leads to death. |
| - When swelling and in the throat (glottis). |
| - On the neck or face. |
| - Facial edema due to the respiratory tract. |
| - When it reaches the respiratory tract. |
| - When it develops quickly and mainly affects the glottis region. |

1. How was the first crisis?

Answers:

- I was a 2-year-old child, my hands were almost always swollen, abdominal pain and cramps

| - Intriguing, I didn't know what it was about. |
| --- |
| - In the first pregnancy, edema in the mouth, hands and genitals. |
| - I do not remember. |
| - Before I got married, I started using contraceptives. |
| - I don't remember. My mother said I was about four years old. |
| - Foot swelling at age 5. |
| - I swelled my face and glottis. |
| - 3 months. |
| - It was moderate, in the arm and hand. |
| - Severe, triggered by the menstrual period. |
| - 1 month. |
| - I have had crises since the first month of life. My mother reports that her hands, feet, arms, legs were swelling and every time she took me to the doctor she was diagnosed with an allergy to some food. |
| - Abdominal. I had my whole childhood and didn't know it was AEH. |
| - After pregnancy, 39 days after birth. |
| - It's been 11 years since I was diagnosed so I don't remember much, but I believe it was swollen on the extremity of my hand or foot. |
| - It was as a child, 2 years old, my hands and feet swelled very often. I had numerous bouts of colic caused by the swelling of the intestinal loops. |
| - At 13 years old. |
| - My mother told me that I was 2 years old, and my hands started to swell, and it hasn't stopped since. |
| - Horrible, I had lip edema. |
| - Intense pain in the stomach and edema on the face. At 14 years old. |
| - Strong, it hit the glottis, days in ICU. |
| - In pregnancy at 16 years of age. Swollen tongue, fingers and genitals. I was diagnosed with pre-eclampsia with blood pressure 12x8. |
| - It was when I was pregnant, on my feet and hands. |
| - At 5 years old. Swelling in the hands. |
| - The first crises appeared in early childhood, but it was diagnosed at the age of 15, with significant facial edema. |
| - Extremely strong. Abdominal. |
| - Swelling in the hands. I went to the emergency room and took corticosteroids. It took about 3 or 4 days to normalize. |
| - When I pulled a tooth, my whole face swelled. |
| - Swelling in one hand at age 17. |
| - I had swellings when I was around 20 years old, and then the frequency increased, it was very difficult because I hadn't yet been diagnosed. |
| - In early childhood, swelling in extremities of the upper limbs. |
| - It was due to a ball hit to the face |
| - As a child, I remember my face swelling after a fall and being unable to see all day. |
| - I was still a child. I do not remember. |
| - The first crisis occurred at the age of 12 with a fall causing a lump on the head with an unusual size. |
| - Eye swelling at age 4 or 5 |
| - Abdominal pain, followed by vomiting and diarrhea. |
| - I don't remember because I was still a child. |
| - During childhood, I had occasional attacks of swelling of my hands and feet. During adolescence, the crises increased, and the diagnosis occurred at the age of 16. |
| - I was 3 years old and according to my mother's report, one of my hands was swollen. My father also had the disease at the time, and we had no diagnosis. |
| - As far as I remember, it was when I was 8 years old and I fell off my bike, deforming my entire face because I cut my chin. |
| - When I got pregnant, at age 20, I started having terrible abdominal attacks, huge swellings on my feet, hands and elbows. |
| - At the age of 18, I had abdominal pain and even had appendix surgery because I didn't know I had the disease. |
| - When I was approximately 3 years old. My lips swelled a lot. |
| - I was a very young child, and I don't remember. |
| - I was a baby. The first time it was on the face. |
| - At 5 years old with abdominal pain and vomiting. |
| - Foot edema. |
| - Everything on my face, mouth, eyes, etc., swelled. |
| - An edema in the genital organ, aged 2 years and 6 months. Today I am 55 years old. |
| - Huge abdominal pain |
| - In adolescence |
| - On hand, at 5 years old |
| - I was a baby, my mother says my hands were swollen, I was crying and vomiting, my father had the same problem. |
| - When I was a child, according to my mother, I had generalized edema all over my body. |
| - 4 years, crisis on the face. |
| - Glottis edema - after application of a desensitizing vaccine for allergic rhinitis. |
| - First attack of swelling in the hand after a fall from a skateboard and it was treated by the orthopedist as a fracture |
| - Eyes. I don't remember because I was a baby, but my mother said it was terrible |
| - It was a swelling in the hand at a year and a half old |
| - Lip and glottis |
| - I don't remember, but it started in childhood with edema on the face, hands and abdomen. |
| - I have had crises since my childhood. Edema in the extremities and face. |
| - 1 year old, swelling in hands. |
| - As a child, around 4 or 5 years old |
| - When he was two years old, his tongue swelled and since then he has had frequent attacks all over his body. |
| - I live in a family that has 18 patients where crises are a constant. I grew up with my father and sister having attacks on the face, internal and external swellings such as the tongue and throat edema. So, I don't know exactly when I witnessed the first crisis. |
|  |
|  |
|  |
|  |
|  |
|  |
|  |
|  |
|  |
|  |
|  |
|  |
